# Diagnosis of autoimmune disease in the setting of immunodeficiency

**DOI:** 10.1186/1710-1492-10-S2-A42

**Published:** 2014-12-18

**Authors:** Lana Rosenfield, Richard Warrington

**Affiliations:** 1 Department of Internal Medicine, University of Manitoba, Winnipeg, Manitoba, Canada; 2 Section of Allergy and Clinical Immunology , Department of Internal Medicine, University of Manitoba, Winnipeg, Manitoba, Canada

## Background

Autoimmune disease and immunodeficiency in the same patient can be a diagnostic dilemma but there is evidence of these conditions occurring simultaneously.

## Case

A 41-year-old male presented with abdominal pain, diarrhea, weakness, and shock. He was diagnosed with cardiac tamponade, underwent pericardial drainage and initiation of broad-spectrum antibiotics.

The patient had a recent admission with bilateral pleural effusions, with negative infectious and malignancy workup. Macrocytic anemia, increased white blood cells, incidental abdominal abscess and splenic infarcts were found during this admission.

Investigations again demonstrated elevated white blood cells (53.8% neutrophils, 12.1% lymphocytes, 29.7% monocytes) and anemia. Autoimmune workup was unremarkable except for presence of atypical ANCA and lupus inhibitor. Bone marrow biopsy was non-specific. He had low IgM and IgG levels suggesting a diagnosis of Common Variable Immunodeficiency (CVID). He received intravenous Solumedrol after his respiratory status deteriorated. He improved and was transitioned to prednisone, but a pathogen was never found.

His positive response to steroids with worsening symptoms with prednisone taper initiation suggested that this was an autoimmune process. He was started on Hydroxychloroquine and intravenous immunoglobulin by Rheumatology. However he was subsequently diagnosed with Chronic Myelomonocytic Leukemia (CMML) based on cytogenetic studies by Hematology. Current treatment includes hydroxyurea and prednisone.

## Discussion

This patient demonstrates the challenge of diagnosing autoimmunity in a patient with low levels of immunoglobulins such as in CVID. How to interpret autoimmune antibody titres in autoimmune conditions is unknown. Most literature describes immunodeficiency being diagnosed after the autoimmune condition [1] and often after immunosuppressant use. Studies on systemic lupus erythematosus and CVID have shown low levels of autoantibodies after immunodeficiency diagnosis.

## Conclusion

This case indicates the dilemma of excluding autoimmune disease in CVID. Although CMML was present, its presentation was atypical and is not known to be associated with CVID.

## Consent

Written informed consent was obtained from the patient for publication of this abstract and any accompanying images. A copy of the written consent is available for review by the Editor of this journal.

## References

[B1] Agarwal S Cunningham-Rundles C Autoimmunity in common variable immunodeficiency Curr Allergy Asthma Re 2009 9 347 52 10.1007/s11882-009-0051-0 PMC2919211 19671377

